# Supplementary material for: Benzotriazine Di-Oxide Prodrugs for Exploiting Hypoxia and Low Extracellular pH in Tumors
Source: Molecules. 2019 Jul 10;24(14):2524. doi: 10.3390/molecules24142524 (PMC6680510; doi:10.3390/molecules24142524)
Supplement: Supplementary file 1 [file molecules-24-02524-s001.pdf]

molecules

Supplementary Material

Benzotriazine di-oxide prodrugs for exploiting hypoxia and low extracellular pH in tumors

Michael P. Hay[1,2], Hong Nam Shin[1], Way Wua Wong[1], Wan Wan Sahimi[3], Aaron T.D. Vaz[1], Pooja Yadav[1], Robert F. Anderson[1,2,3], Kevin O. Hicks[1,2] and William R Wilson [1,2,*]

1 Auckland Cancer Society Research Centre, School of Medical Sciences, Faculty of Medical and Health Sciences, University of Auckland, Auckland 1142, New Zealand.
2 Maurice Wilkins Centre for Molecular Biodiscovery, University of Auckland, Symonds St, Auckland 1142, New Zealand.
3 School of Chemical Sciences, University of Auckland, Auckland 1142, New Zealand
* Correspondence: wr.wilson@auckland.ac.nz; Tel.: +64-9923-6883

Table of Contents:

Table S1: Cytotoxicity of chlorambucil, SN30000 and 4a against UT-SCC-74B …Pg 2

Figure S1: pKa dependence of selectivity of weak acids for acidosis. …Pg 2

Figure S2: pH dependence of absorbance of the radical anion of 7a …Pg 3

Figure S3: Decay kinetics of the radical anion of 7a at pH 7.0 …Pg 3

Figure S4: Oxygen dependence of the decay of the radical anion of 7a at pH 7.0 …Pg 4

Figure S5: EPR spectrum of one-electron reduced **7a** in the presence of DMSO …Pg 5

Figure S6: Growth of SiHa cells after exposure to pHe 6.5 or 7.4 under oxia, hypoxia or anoxia …Pg 6

Figure S7: Protein binding of 7a in mouse plasma and FBS …Pg 6

Figure S8: IC50 of SN30000, 4a and 7a in MDCK-II cells with forced expression of BCRP or Pgp …Pg 7

Figure S9: Effect of phenylpyruvate on cytotoxicity of 4a against SiHa cells …Pg 8

Table S1. Cytotoxicity of chlorambucil, SN30000 and BTO acid **4a** against UT-SCC-74B cells by IC_{50} assay after 4 h exposure at pHe 7.4 or 6.5 under oxic (20% O_2) or anoxic (<0.01% O_2) conditions.

Cmpd	N[a]	pHe	Oxic IC_{50} (μM)	Anoxic IC_{50} (μM)	ACR[b]	PCR[c] oxic	PCR[c] anoxic	TME ratio[d]
CHL (**1**)	4	7.4	6.34 ± 0.88	5.05 ± 0.38	1.26 ± 0.20			
		6.5	0.8 ± 0.17	0.85 ± 0.06	0.94 ±0.21	7.93 ± 2.01	5.94 ± 0.61	7.46 ± 1.16
SN30000	3	7.4	477 ± 65	5.92 ± 2.42	80.6 ± 34.7			
(**2**)		6.5	190 ± 42	6.29 ± 1.79	30.2 ± 10.9	2.51 ± 0.65	0.94± 0.47	75.8 ± 23.9
**4a**	4	7.4	863 ± 66	225 ± 26	3.84 ± 0.53			
		6.5	297 ± 74	97.6 ± 12.3	3.04 ± 0.85	2.91 ± 0.76	2.31 ± 0.39	8.84 ± 1.30

Footnotes: [a] Number of experiments. [b] Anoxic cytotoxicity ratio = oxic IC_{50}/anoxic IC_{50}. [c] pH cytotoxicity ratio = IC_{50} at pHe 7.4/ IC_{50} at pHe 6.5. [d] Overall ratio = oxic IC_{50} pHe 7.4 / anoxic IC_{50} pHe 6.5. Values are means and errors are SEM. For ratios, the errors are root mean square SEM.

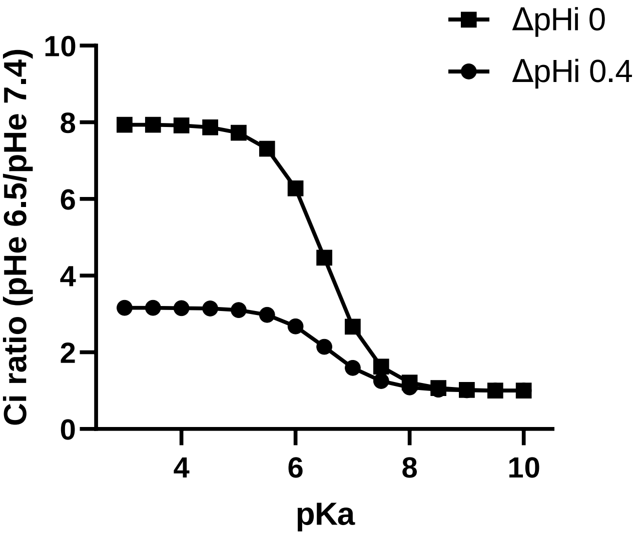

Figure S1. pKa dependence of selectivity of weak acids for extracellular acidosis. The ordinate is the ratio of intracellular concentration at extracellular pH (pHe) 6.5 to that at pHe 7.4. Squares represent the values under the assumption that pHi is 7.1 and is independent of pHe (ΔpHi, the difference in pHi values at pHe 7.4 and 6.4 is zero, i.e. cells fully control pHi under acidosis) or that pHi = 7.3 at pHe 7.4 and pHi = 6.9 at pHe 6.5 (ΔpHi = 0.4) as in the present study.

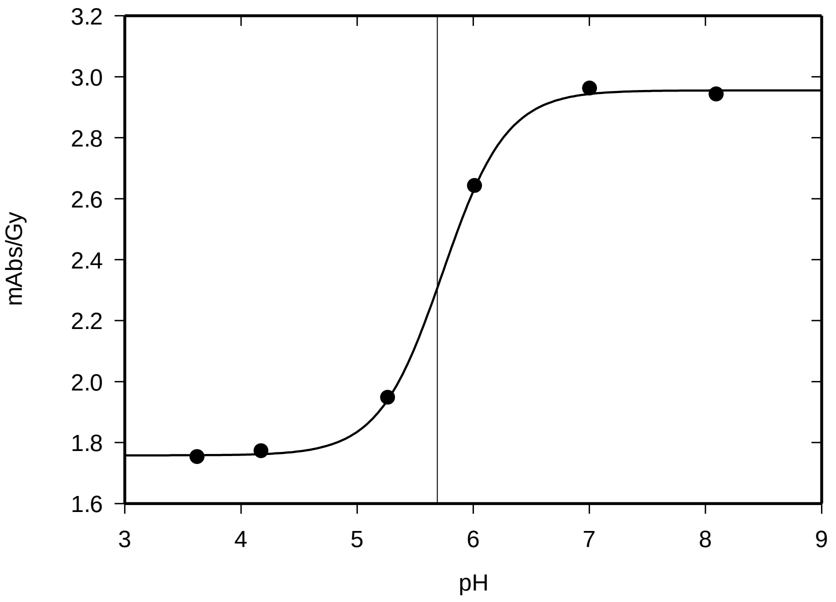

Figure S2. Effect of pH on the change in radical absorption at 490 nm upon one-electron reduction of **7a** (150 mM) by the $CO_2^{\bullet -}$ radical anion following pulse radiolysis (3 Gy in 200 ns) of N_2O-saturated solutions containing sodium formate (0.1 M) and buffers (10 mM). Data points, obtained ca. 20 ms after the pulse, are fitted to a sigmoidal curve yielding a pKa value of 5.69±0.15.

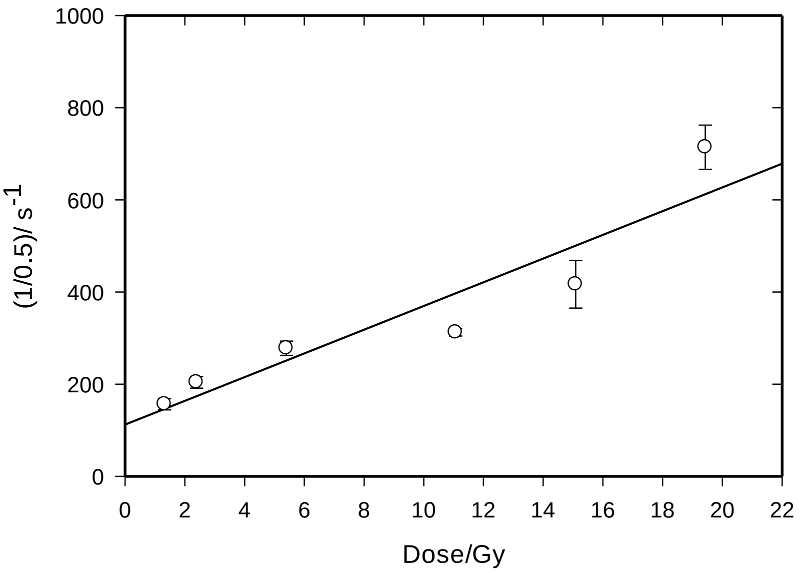

Figure S3. The radical anion was generated by pulse radiolysis of N_2O-saturated solutions containing **7a** (150 mM), sodium formate (0.1 M), phosphate buffer (10 mM), pH 7.0. The plot shows the dependence on increasing radiation dose (radical concentration) of the reciprocal of the first half-life of decay of the radical anion of **7a** measured at 490 nm. The intercept of the plot on the ordinate gives k_1= ln 0.5 x 1/($t_{0.5}$), from which the first order rate constant k_1 is estimated as 78 ± 4 s^{-1}, while the gradient gives the second-order rate constant, k_2 = 3.78 ± 0.73 x 10^7 M^{-1} s^{-1}.

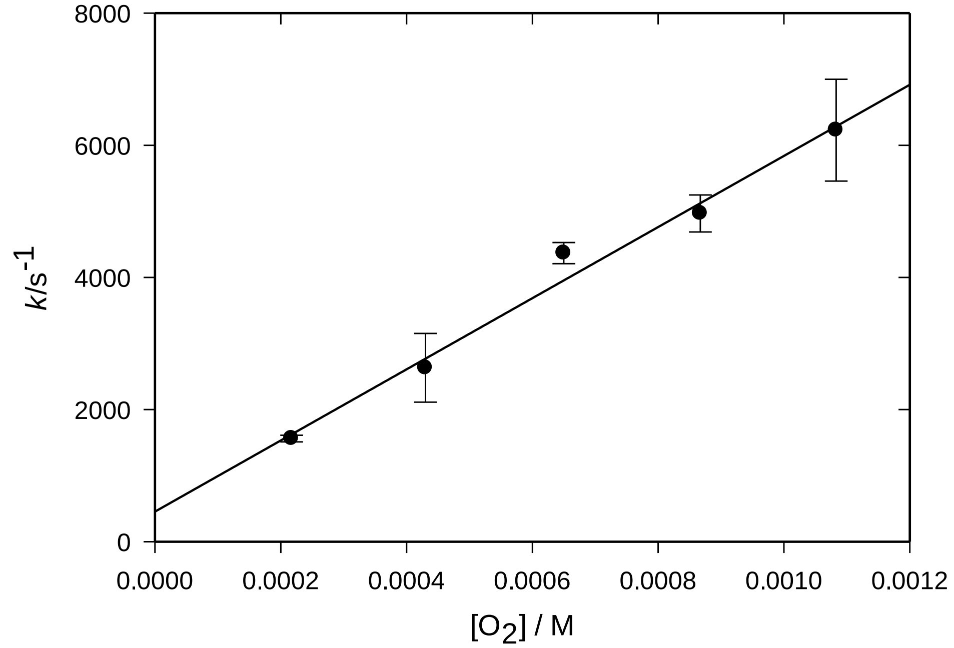

Figure S4. Dependence of the first-order rate constant measured at 490 nm for the decay of the radical anion of **7a** at pH 7.0, *k*, on the concentration of O_2 in solution following pulse radiolysis (10 Gy in 200 ns). Solutions contained **7a** (0.5 mM), sodium formate (0.1 M), phosphate buffer (5 mM), pH 7.0 and saturated with mixtures of O_2/N_2O. The second order rate constant, kO_2, calculated from the gradient of the plot, $kO_2 = 5.39 \pm 0.40 \times 10^6$ M^{-1} s^{-1}.

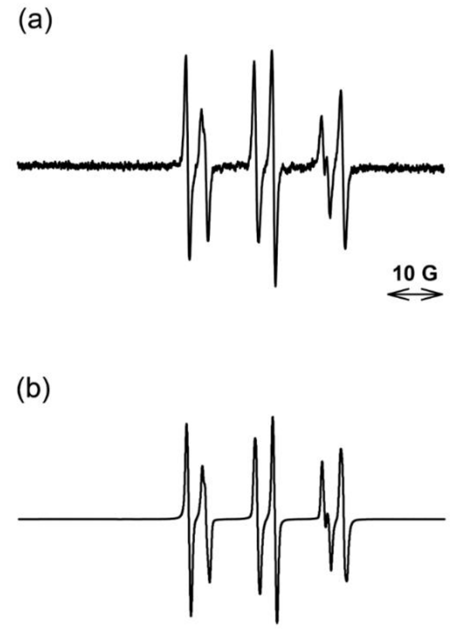

Figure S5. (a) EPR spectrum (average of 100, 2 min scans) obtained upon reduction of **7a** (17 mM) by sPOR protein (7 ng mL^{-1}) in the presence of DMSO (2 M). The anaerobic solution at 310 K contained DETAPAC (100 mM), SOD (300 units mL^{-1}), catalase (1500 units mL^{-1}), glucose-6-phosphate dehydrogenase (13 units mL^{-1}), glucose-6-phosphate (10 mM) and NADPH (1 mM). **(b)** Simulation of the spectrum fitted to the combination of two species, (i) a C-centred radical and (ii) an aryl-type radical, in the ratio 0.65:0.35. The hyperfine coupling constants (HFC) of (i) and (ii) are aH 3.7 G, aN 16.5 G and aH 4.3 G , aN 15.9 G respectively,. r = 0.980.

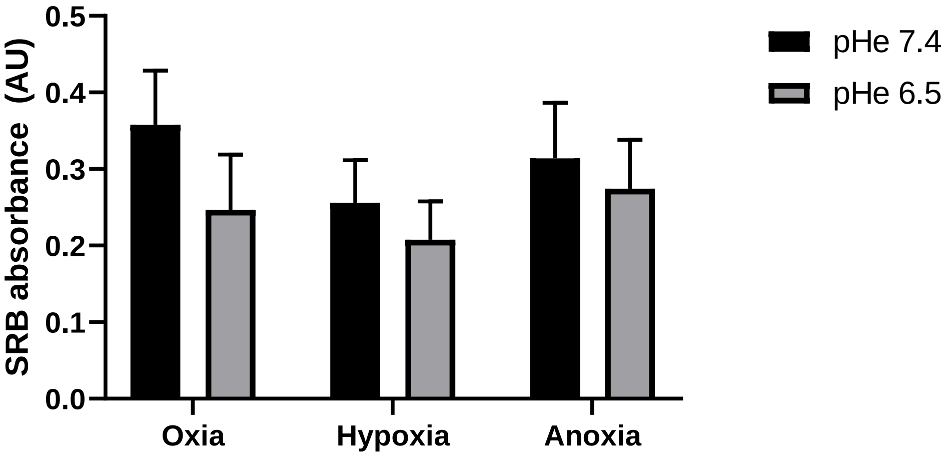

Figure S6. Growth of control (non-drug-treated) SiHa cells following seeding 1500 cells/well in 96-well plates, exposure to hypoxia (0.2% O_2) for 24 h or anoxia (<0.01% O_2) for 6 h or continuous growth under oxia (20% O_2), followed by growth in fresh medium at pHe 7.4 for 5 days under oxia before staining with SRB. Values are mean and errors are SEM for 6 experiments. Differences between pHe values and gas phase O_2 concentrations were not significant ($P < 0.05$) by two-way ANOVA.

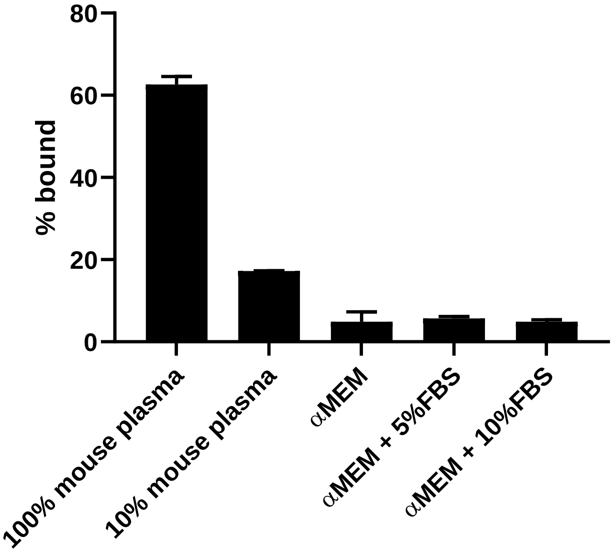

Figure S7. Protein binding of **7a** in plasma from NIHIII mice and fetal bovine serum (FBS) assessed by equilibrium dialysis using a 12-14KDa cut-off dialysis membrane (HTDialysis, Gales Ferry, CT) as described by Banker et al., *J. Pharm. Sci.* 92, 967-974. Briefly, 100 μL samples of undiluted plasma, plasma diluted in PBS, and αMEM with or without FBS containing **7a** at 100 μM were dialysed against 100 μL PBS or αMEM (pH 7.4) in an incubator (Innova-42, New Brunswick Scientific, US) at 37°C with shaking at 80 rpm for 6 h. Proteins were precipitated with 3 vol of ice-cold MeCN, the centrifuged supernatants were analysed by HPLC as described in Section 4.12.

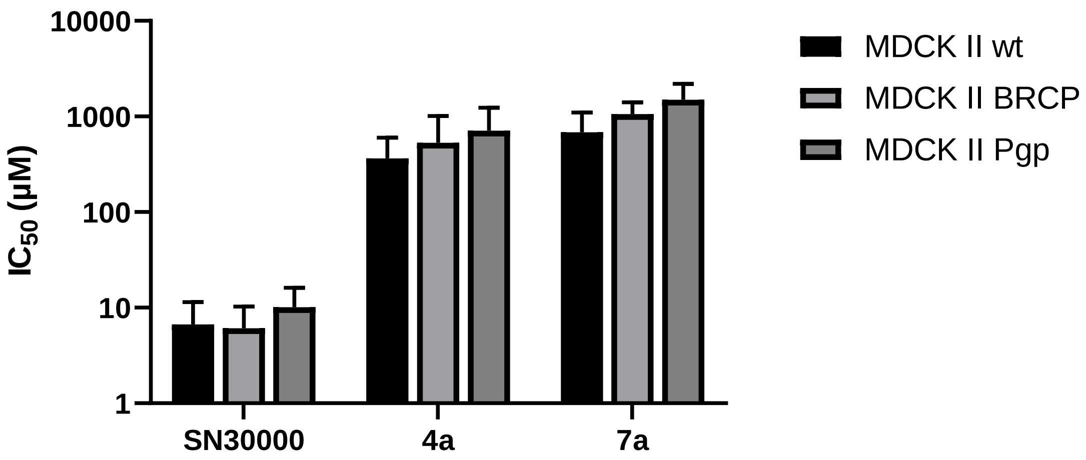

Figure S8. IC_{50} values of SN30000 and two BTO acids (**4a** and **7a**) in MDCK-II cells with forced expression of BCRP or Pgp. The cell lines were gifts from A/Professor James Paxton, University of Auckland. Cells were seeded at 300 cells/well in DMEM with 10% FBS in 96 well plates in an anaerobic chamber, allowed to attach for 2 h then exposed to drugs for 4 h under anoxia. Cells were then washed with fresh medium and grown for 5 days under aerobic conditions before staining with SRB. Values are mean ± SEM from 2 separate experiments.

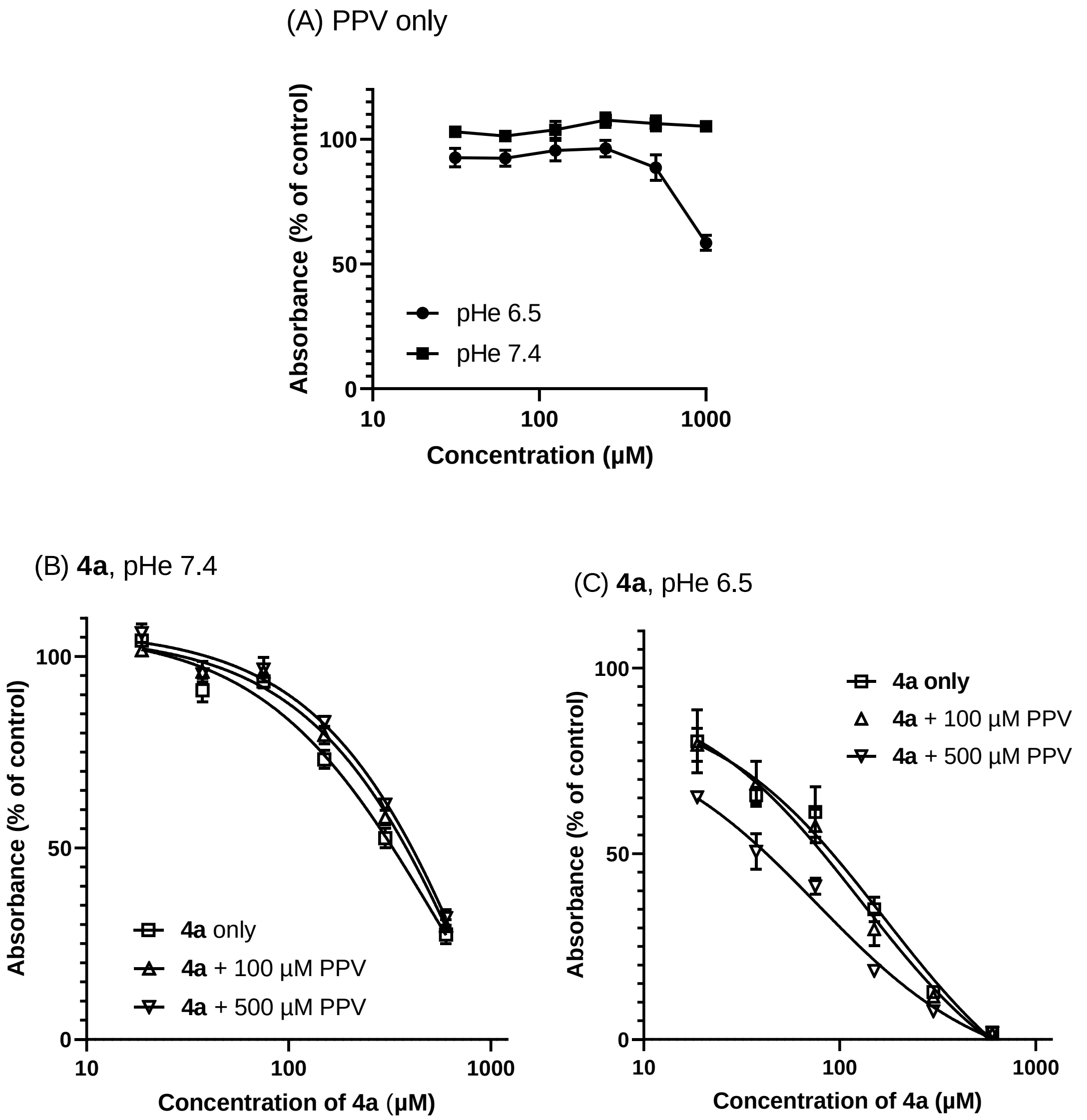

Figure S9. Effect of phenylpyruvate (PPV, 100 or 500 μM) on proliferation of SiHa cells following 4 h exposure under anoxia to PPV alone (A) or with cmpd **4a** at pHe 7.4 (B) or pHe 6.5 (C). 3000 cells/well were seeded in 96 well plates, and IC_{50} values determined as in Figure S6. Each data point is mean ± SEM for three replicate cultures in the same experiment. There is some evidence in panel B for an effect of 500 μM PPV on the cytotoxicity of **4a** at pHe 6.5, but PPV alone showed selective cytotoxicity at pHe 6.5 relative to pHe 7.4 (panel A) which makes this difficult to interpret. No effect of PPV was observed under non-toxic conditions (pHe 7.4, or 100 μM PPV at pHe 6.5).
